# Functional Coating Effects of Silver Diamine Fluoride (SDF) on Artificial Caries Lesions: A Microhardness-Based Evaluation

**DOI:** 10.3390/ma18163889

**Published:** 2025-08-20

**Authors:** Mohammed H. Alshamrani, Reem A. Alajlan, Waad E. Alsaadi, Amjad M. Alabdulmohsen, Munira Abuthnain, Carlos Fernando Mourão, Adam Lowenstein

**Affiliations:** 1 Pediatric Dentistry and Orthodontic Department, College of Dentistry, King Saud University, Riyadh 11545, Saudi Arabia; malshamrani2@ksu.edu.sa (M.H.A.); ralajlan@ksu.edu.sa (R.A.A.); walsaadi@ksu.edu.sa (W.E.A.); amjad@ksu.edu.sa (A.M.A.); 2 College of Dentistry, King Saud University, Riyadh 11545, Saudi Arabia; munirabuthnain@gmail.com; 3 Department of Basic and Clinical Translational Sciences, Tufts University School of Dental Medicine, Boston, MA 02111, USA; carlos.mourao@tufts.edu

**Keywords:** silver diamine fluoride (SDF), primary teeth, enamel remineralization, caries prevention, dental caries, minimally invasive dentistry

## Abstract

**Background**: Dental caries is a prevalent dental problem affecting primary and permanent teeth. Early demineralization of enamel lesions can be reversed through remineralization. Many studies have focused on caries prevention and disease progression arrest using silver diamine fluoride (SDF). No in vitro studies have compared the remineralization effects of different 38% SDF solutions on artificially demineralized enamel lesions. This study aimed to compare the remineralization potential of three commercial 38% silver diamine fluoride formulations on artificial enamel lesions in primary teeth using a pH cycling model. The hypothesis was as follows: different commercial SDF formulations would exhibit varying remineralization effects, as measured by surface microhardness, due to potential differences in their compositions. **Materials and Methods**: In this study, 75 primary molars were randomized into five groups (N = 15): I: baseline, II: SDF Riva Star Aqua^®^ 38%, III: Riva Star^®^ 38%, IV: SDF Advantage Arrest^®^ 38%, and V: control. Artificial caries were created by submerging teeth in 10 mL of demineralization solution (pH 4.5) for three days in a light-resistant container, ensuring distinct visual changes in the enamel as per the International Caries Detection and Assessment System (ICDAS level 2). After pH cycling, all samples underwent a standardized Vickers microhardness test (VMHT) with a 50 g load for 15 s. Data were analyzed using one-way ANOVA and Tukey’s post hoc test, with a significance level set at *p* ≤ 0.05. **Results**: The one-way ANOVA test indicated a significant difference in microhardness among the groups (SDF Riva Star Aqua, SDF Riva Star, and SDF Advantage Arrest), with an F-value of 167.73 and *p* < 0.001. The post hoc Scheffé test showed that SDF Riva Star Aqua and SDF Riva Star were not significantly different (*p* = 0.388). However, SDF Advantage Arrest had a significantly higher mean microhardness compared to both groups (*p* < 0.001). Overall, these results show that SDF Advantage Arrest leads to greater microhardness than SDF Riva Star Aqua or SDF Riva Star. **Conclusions**: SDF Advantage Arrest showed superior performance among the SDF-treated groups, significantly increasing microhardness compared to SDF Riva Star Aqua and SDF Riva Star. This suggests that SDF Advantage Arrest offers enhanced remineralization and structural strengthening, making it the most effective option for managing demineralized primary teeth. Future research should investigate the long-term performance and mechanisms of these treatments to optimize clinical protocols for preserving primary tooth integrity.

## 1. Introduction

Dental caries is considered one of the most prevalent dental problems affecting both primary and permanent dentitions. It starts as a white, chalky lesion on the enamel surface, clinically known as a decalcified or demineralized lesion. In addition, the manifestation of dental caries can range from a simple demineralization lesion (non-cavitated) to a complete destruction and loss of tooth structures (cavitated lesions). Any alteration in the balance between the host and the oral flora can result in dental caries. Normally, in the oral cavity, the saliva is saturated with calcium and phosphate ions in addition to the natural pH, which ranges between 6.5 and 7.4, which is considered a balanced environment within the oral cavity. However, any alterations in the oral environment, either due to the presence of carcinogenic bacteria, known as Streptococcus mutans, or the presence of acids from food or drinks, can change the natural pH to below five and shift the natural balance toward demineralization [1,2]. At the early stages of dental caries, demineralization of the enamel lesions can be reversed by an active mechanism known as remineralization. It is an active natural process to repair non-cavitated enamel lesions by deposing calcium, phosphate, and fluoride ions into the porous demineralized enamel surface [1,3]. This process can be achieved by the application of various dental materials, such as fluoride varnish (5%NaF), silver diamine fluoride (38% SDF), and glass ionomer fissure sealant (GIC).

Furthermore, many studies have been directed toward caries prevention and arresting disease progression by using silver diamine fluoride (SDF) [4]. SDF, 5% F 44,800 ppm F, is a colorless alkaline solution with a pH value between 9 and 10 [5]. It contains 25% silver, 5% fluoride, and 8% ammonia [6]. SDF combines the antimicrobial effects of silver with the remineralizing effects of fluoride [7]. SDF interacts with hydroxyapatite to form calcium fluoride and silver phosphate, which enhances the mineral content of dental hard tissue. Thus, surface microhardness significantly improves, while the carious lesion depth decreases [8,9]. SDF has been used since 1969 to arrest carious lesions in young children and root caries in aged people. It has also been used to prevent pit and fissure caries, sterilize infected root canals, and treat tooth hypersensitivity [10]. In 2014, the United States Food and Drug Administration approved its use as a dentinal hypersensitivity treatment for adults, and off-label use for caries arrest is permissible and appropriate for patients [11]. SDF is a safe, simple, painless, noninvasive, inexpensive, effective, and efficient solution that is almost twice as effective as fluoride varnish for caries arrest [12]. Fung et al. conducted a randomized clinical trial study that showed that 38% SDF is more effective in arresting dentin carious lesions than 12% SDF concentration with the biannual application [13]. The American Academy of Pediatric Dentistry established guidance and an evidence-based recommendation that supports the application of 38% SDF and its off-label use for carious lesion arrest in primary dentition [14,15]. A systematic review suggested that SDF is more effective in anterior teeth than in posterior teeth and that its success rates seem independent of the time of application. The use of SDF is not recommended for teeth with suspected pulpal involvement [10].

Dark staining is a frequent side effect following the application of SDF, and it forms due to the formation of silver phosphate and silver sulfide precipitate on the carious dentin [16]. Dark staining can be reduced by using potassium iodide, which reacts with the remaining free silver ions to form silver iodide and tripotassium phosphate, forming a white powder [10]. A study by Zhao et al. showed that potassium iodide reduces the discoloration caused by SDF [17]. However, Roberts et al. conducted a systematic review that showed a poor level of evidence supporting the use of potassium iodide to reduce the discoloration caused by SDF [18].

Based on a recent systematic review, sealants, fluoride gels, and varnishes have contributed to preventing new caries lesions and arresting them at early stages [19]. A study that compared SDF and fluoride varnish showed that SDF was significantly better than fluoride varnish at 18 and 30 months. Moreover, another study that compared SDF with glass ionomer cement (GIC) concluded that GIC was better than SDF at 12 months. However, the difference was not statistically significant. Both studies are weak because they are based on only one trial each [15].

Although various remineralizing dental materials are available, there is inconclusive evidence on the superiority of a specific material over the others in primary teeth. Few in vitro studies have investigated the prevention of demineralization of artificial enamel caries with different types of fluoride varnishes in comparison to 38% SDF [2,20].

Various remineralizing agents are available, and SDF is increasingly favored for its dual antimicrobial and remineralizing actions [5,7,15,21]. The rationale for comparing specific commercial SDF formulations stems from potential, yet often proprietary, differences in their chemical makeup, such as stabilizers, pH, and viscosity, which may influence silver ion availability and ultimately affect remineralization efficacy. This study is novel as it employs a pH cycling model to assess microhardness changes in primary molar enamel, providing a robust in vitro simulation of caries dynamics [3,20,22]. The objective of this study was to evaluate the remineralization effects of three different commercial 38% SDF solutions on artificial enamel lesions in primary molars. The null hypothesis was that there would be no difference in the surface microhardness of enamel treated with the different SDF formulations after pH cycling.

Accordingly, this study aimed to compare the effects of different commercially available 38% silver diamine fluoride solutions on the microhardness of artificially demineralized enamel lesions in primary molars following pH cycling. It was hypothesized that significant differences would exist in the remineralization potential among the treatment groups.

## 2. Materials and Methods

An in vitro study was conducted in the Physical Laboratory of the College of Dentistry, King Saud University (KSU), Riyadh, Saudi Arabia. The study adhered to the approved research protocol and the ethical guidelines of the King Saud University Institutional Review Board (IRB), as referenced under number 24/1301/IRB.

### 2.1. Sample Size

Based on previous studies, the sample size was calculated using G*power software (v 3.1.9.7) with an effect size of 0.60, an alpha level of 5%, and a power of 85%. Analysis indicated that 75 primary molars, with 15 from each group, are necessary to detect significant differences among the study groups [22].

### 2.2. Study Sample Standardization and Exclusion Criteria

**Figure 1:**
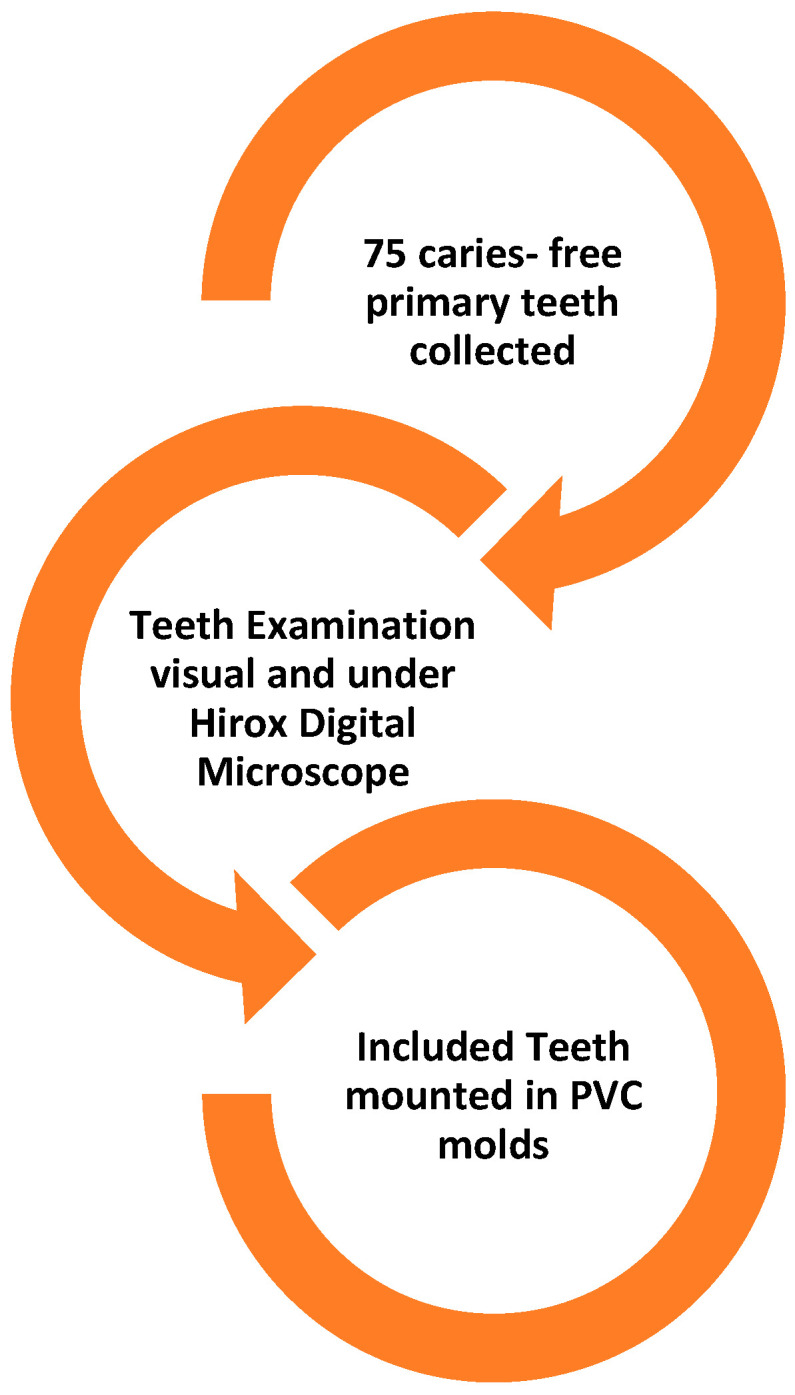
Sample Collection, inspection, inclusion, and preparation.

A total of 75 sound primary molars, whether exfoliated or extracted as part of an orthodontic treatment plan, were collected from the clinics within the Pediatric Dentistry and Orthodontics (POS) department at DUH, KSU. Verbal consent was obtained from the patients and parents before collection. Exclusion criteria included teeth with enamel demineralization, defects, cracks, developmental anomalies, or visible/detectable caries, according to the International Caries Detection and Assessment System (ICDAS). The teeth were thoroughly cleaned and polished using a fluoride-free polishing paste applied with rubber prophylactic cups. They were then carefully examined by a trained general dental practitioner, both visually and under a digital microscope (Hirox HRX-01, Hirox, Hamburg, Germany), to assess their eligibility for inclusion. Then, to ensure that only the crown was visible, each tooth was positioned vertically in a self-curing clear orthodontic resin block using polyvinyl chloride (PVC) molds that were 2.80 mm in diameter and 10 mm in thickness. Following a sequential polishing procedure using an automated machine that included 400-, 800-, and 1200-grit sandpaper, the buccal enamel surface was polished and flattened by 0.5 mm at the most convex portion of the enamel. Enamel blocks were randomly allocated to the three treatment groups using a computer-generated sequence. Allocation concealment was ensured by having an independent operator, who was not involved in specimen preparation or outcome assessment, manage the randomization list. The flowchart of the study design is illustrated in Figure 1.

### 2.3. Artificial Caries Preparation

All included teeth, except those in the baseline group, underwent subsurface enamel lesion formation (white spot lesions, WSLs) by being submerged individually in 10 mL of a demineralization solution (pH 4.5) for three days inside a light-resistant container. After this process, the level of artificial caries was carefully evaluated to ensure the presence of distinct visual changes in the enamel under both dry and wet conditions (ICDAS level 2) [23]. The demineralization solution consisted of 2.2 mmol/L calcium chloride (CaCl_2_), 2.2 mmol/L monosodium phosphate (NaH_2_PO_4_·7H_2_O), and 50 mmol/L acetic acid, with the pH adjusted to 4.4 with 1 mol/L KOH. The remineralization solution contained 1.5 mmol/L calcium chloride, 0.9 mmol/L monosodium phosphate, and 150 mmol/L KCl, adjusted to pH 7.0 [20,22]. The compositions of the demineralization and remineralization solutions used are detailed in Table 1 [22].

### 2.4. Study Groups

The included samples were randomly divided into five equal groups by a study-independent operator (**I**: baseline, **II**: SDF Riva Star Aqua^®^ 38%, **III**: Riva Star^®^ 38%, **IV**: SDF Advantage Arrest^®^ 38%, and **V**: control group), each comprising 15 samples (N = 15) (Table 2). The three commercially available 38% SDF preparations used were Riva Star Aqua^®^ (SDI Limited, Bayswater, VIC, Australia), Riva Star^®^ (SDI Limited, Bayswater, VIC, Australia), and Advantage Arrest^®^ (Elevate Oral Care, West Palm Beach, FL, USA). According to manufacturer specifications, all products contain 38% (*w*/*v*) silver diamine fluoride, with other components such as stabilizers being proprietary.

### 2.5. Treatment Protocol

A single application of SDF was performed for 2 min, a standard protocol justified by its common use in previous studies [22].

#### 2.5.1. Group I (Baseline)

The control group consisted of 15 specimens that were left untreated and stored in artificial saliva for 24 h. There was no artificial caries preparation in this group. Before pH cycling, the specimens were rinsed with deionized water for one minute.

#### 2.5.2. Group II (SDF RIVA STAR AQUA^®^ 38%)

After artificial caries preparation, SDF was applied to the exposed enamel for 2 min with a micro-brush. The teeth were then washed with 10 mL of deionized water for 30 s, dried with compressed air, and stored in artificial saliva for 24 h. Afterward, the teeth were rinsed with deionized water for 1 min before pH cycling.

#### 2.5.3. Group III (SDF RIVA STAR^®^ 38%)

SDF was applied with a micro-brush to the exposed enamel for 2 min after artificial caries preparation. The teeth were washed with 10 mL of deionized water for 30 s, dried with compressed air, and stored in artificial saliva for 24 h. Subsequently, the teeth were rinsed with deionized water for 1 min before pH cycling.

#### 2.5.4. Group IV: SDF (SDF Advantage Arrest^®^ 38%)

After artificial caries preparation, SDF was applied with a micro-brush to the exposed enamel for 2 min. The teeth were washed with 10 mL of deionized water for 30 s, dried with compressed air, and stored in artificial saliva for 24 h. The teeth were then rinsed with deionized water for 1 min before pH cycling.

#### 2.5.5. Group V: Control (Placed in Artificial Saliva for 24 h)

After artificial caries preparation, the teeth were left untreated and stored in artificial saliva for 24 h. Before pH cycling, the teeth were rinsed with deionized water for 1 min.

### 2.6. pH Cycling

1. Except for the baseline group, all specimens underwent an 8-day pH cycling process, consisting of 7 days of de/remineralization cycles followed by 1 day of remineralization. The 7-day pH cycling protocol, followed by 1 day of pure remineralization, was chosen to simulate the early-phase dynamics of lesion formation and repair in the oral environment. This regimen is established in validated in vitro models for assessing the early effects of remineralizing agents. The 7-day duration specifically allows for capturing the initial remineralization response, a timeframe supported by similar recent studies [20,22].
2. The demineralization (pH 4.4) and remineralization (pH 7) cycles involved immersing the specimens in 10 mL of demineralization solution for 4 h, followed by immersion in 10 mL of remineralization solution (artificial saliva) for 20 h.
3. This cycle was repeated daily for seven consecutive days, with fresh solutions used for each cycle.
4. After each cycle, specimens were thoroughly rinsed with deionized water before commencing the next cycle.
5. On day 8, the specimens were immersed in the remineralization solution (pH 7) for 24 h.

### 2.7. Vickers Microhardness Test (VMHT)

Following the pH cycling process, all samples were subjected to standardized VMHT (Highwood DMH 7, Model HWMMT-X7, TTS Unlimited, Osaka, Japan) under a 50 g load for 15 s. The outcome assessor performing the Vickers microhardness testing was blinded to the group allocation of the specimens to prevent bias.

### 2.8. Statistical Analysis

Data were analyzed to confirm normality using the Shapiro–Wilk test. One-way analysis of variance (ANOVA) was used to compare the mean microhardness values among the three groups, followed by the Scheffé post hoc test for pairwise comparisons. The effect size was calculated using partial eta squared. Outliers were identified using boxplots, but none were removed as they were deemed to be within the plausible range of biological variation. The significance level was set at *p* ≤ 0.05 within all tests. Descriptive statistical analysis was performed using the SPSS program (IBM SPSS Statistics 29, IBM, Armonk, NY, USA).

## 3. Results

The primary descriptive analysis showed distinct differences in the mean values of the five groups under investigation (**I**: baseline, **II**: SDF Riva Star Aqua^®^ 38%, **III**: Riva StAR^®^ 38%, **IV**: SDF Advantage Arrest^®^ 38%, and **V**: control group), each comprising 15 samples (N = 15). The baseline group exhibited the highest mean values (M = 469.32, SD = 1.01), indicating a markedly greater value than the other groups. By contrast, the control group demonstrated the lowest mean (M = 192.56, SD = 2.24). The three SDF-based groups displayed intermediate means, with Group **IV** (Advantage Arrest) achieving a somewhat higher mean (M = 333.59, SD = 1.82) than Groups **II** (Riva Star Aqua; M = 323.32, SD = 1.79) and **III** (Riva Star; M = 322.38, SD = 1.97), which were nearly identical to each other, as seen in Table 3.

All five groups showed relatively low within-group variability, as indicated by small standard deviations and correspondingly tight 95% confidence intervals. The baseline group’s 95% CI [468.60, 470.04] and the control group’s CI [191.33, 193.80] suggest consistent measurements within each condition. Additionally, the overlapped confidence intervals for Groups **II** and **III** reflect their statistical similarity. In contrast, Group **IV**’s confidence interval did not overlap with those of Groups **II** or **III**, indicating a distinct intermediate position.

The data suggest clear stratification among groups when employing multiple comparison techniques (as indicated by categorical letters, such as ‘g’ for baseline, ‘c’ for Groups **II** and **III**, ‘d’ for Group **IV**, and ‘a’ for Group **V**).

Baseline remained uniquely high (g), the two Riva Star-treated groups clustered together (c), Advantage Arrest occupied its own intermediate tier (d), and the control condition stood alone at the lower extreme (a).

The ANOVA revealed a statistically significant difference among the treatment groups (*p* < *0*.001), with a large effect size. The one-way ANOVA test showed a statistically significant difference in microhardness among the three groups (**II** = SDF Riva Star Aqua, **III** = SDF Riva Star, and **IV** = SDF Advantage Arrest), with an F-value of 167.73 and *p* < 0.001, as seen in Table 4.

The post hoc Scheffé comparisons revealed that Groups **II** (SDF Riva Star Aqua) and **III** (SDF Riva Star) did not differ significantly from each other (*p* = 0.388), indicating that they yielded statistically similar microhardness values. In contrast, Group **IV** (SDF Advantage Arrest) showed a significantly higher mean microhardness compared to both Groups **II** and **III** (*p* < 0.001 for both comparisons), as seen in Table 5.

The homogeneous subset analysis further supported this pattern: Groups **II** and **III** clustered together in one subset, while Group **IV** stood alone in a separate, higher subset, as seen in Table 6.

Overall, these results demonstrate that SDF Advantage Arrest (Group **IV**) led to significantly greater microhardness than either SDF Riva Star Aqua (Group **II**) or SDF Riva Star (Group **III**), which were similar.

## 4. Discussion

The findings of this study demonstrate significant differences in microhardness among the tested groups, providing insights into the effects of different treatments on primary teeth. The baseline group, representing untouched, intact primary teeth, exhibited the highest microhardness values. This aligns with the literature emphasizing the natural resilience of unaltered enamel and dentin in primary teeth [5]. These results underscore the critical role of maintaining the integrity of primary teeth to optimize their natural strength and resistance to external stressors [24].

In contrast, the control group, which underwent acid-induced artificial caries and was subsequently stored in artificial saliva without treatment, demonstrated the lowest microhardness values. This finding is consistent with studies highlighting that demineralization significantly weakens the tooth structure, and artificial saliva alone provides insufficient remineralization to restore hardness [25]. The pronounced reduction in hardness observed in the control group highlights the necessity of active remineralization strategies to mitigate the effects of acid-induced demineralization [25].

Among the SDF-treated groups, SDF Advantage Arrest (Group **IV**) exhibited significantly higher microhardness values compared to both SDF Riva Star Aqua (Group **II**) and SDF Riva Star (Group **III**). The superior performance of Advantage Arrest may be attributable to differences in its formulation. While the exact compositions are proprietary, factors such as pH, viscosity, and the presence of specific stabilizers can impact silver ion availability and its interaction with the enamel surface, potentially leading to more effective remineralization. These findings are consistent with previous studies, although direct quantitative comparisons are challenging due to methodological variations [5,15,21]. The near-identical performance of Groups **II** and **III** reflects findings in the literature that report minimal differences in outcomes when using similar fluoride concentrations and application protocols [21].

Post hoc Scheffé analysis further confirmed these relationships, showing that Groups **II** and **III** were statistically similar, while Group **IV** stood apart as significantly superior. The homogeneous subset analysis reinforced this pattern, placing Groups **II** and **III** in a single subset and Group **IV** in a separate, higher subset. The stratification of results suggests that SDF Advantage Arrest 38% has distinct advantages over the other SDF formulations in enhancing the microhardness of primary teeth after demineralization.

The low within-group variability and tight confidence intervals observed in all groups lend reliability to these findings. The overlap of confidence intervals for Groups **II** and **III** reflects their similarity, while the distinct interval for Group **IV** emphasizes its superior performance. As expected, the baseline and control groups had no overlap with other groups, reinforcing their positions as the hardest (untouched) and softest (acid-demineralized without treatment) groups, respectively.

These results highlight the importance of using effective remineralization agents like SDF to counteract the effects of demineralization and enhance the structural integrity of primary teeth [26]. Advantage Arrest demonstrated superior performance, suggesting that its formulation offers unique benefits for strengthening teeth following acid-induced demineralization.

Although this is an in vitro study, the results suggest that the choice of commercial SDF product could influence clinical outcomes for early enamel lesions in primary teeth. The enhanced remineralization seen with Advantage Arrest may be beneficial in a clinical setting; however, factors such as the presence of biofilm, saliva flow, and patient compliance must be considered [3,5,21].

The use of a Vickers microhardness test exclusively presents a limitation. The test assesses surface-level changes and does not provide information on subsurface mineralization. Complementary methods like transverse microradiography or scanning electron microscopy would offer a more comprehensive analysis [3,20]. Another limitation is as follows: the 7-day experimental period reflects an early-stage effect, and these findings cannot be extrapolated to long-term clinical performance without further investigation [21,22]. Lastly, the absence of a natural oral biofilm and the short duration of the study limit the direct clinical extrapolation of these results [3,5,22].

Given the critical role of primary teeth in maintaining oral function and guiding the development of permanent teeth, these findings have important clinical implications. Advantage Arrest could serve as the preferred choice for managing demineralized primary teeth at risk of caries progression.

Although the immediate microhardness (MH) observed with SDF treatments may appear lower than other interventions, such as fluoride varnish, long-term research is likely to reveal an increase in MH over time. SDF forms a protective layer of silver and fluoride on the tooth surface, creating a barrier against further demineralization while maintaining a reservoir of fluoride ions. This layer, coupled with SDF’s potent antibacterial properties, reduces the cariogenic environment and facilitates ongoing remineralization over extended periods [15]. As fluoride ions are gradually released and incorporated into the tooth structure, deeper mineral deposition and strengthening are expected to occur, improving mechanical properties and resulting in higher microhardness over time [5]. Long-term studies that simulate dynamic oral conditions and prolonged fluoride release are essential to fully understand these effects and confirm the enduring benefits of SDF in enhancing and preserving the structural integrity of both primary and permanent teeth.

Future studies should investigate the long-term effects of these treatments under dynamic oral conditions and their performance on various stages of caries progression. Additionally, understanding the underlying mechanisms behind Advantage Arrest’s superior performance can inform the development of more effective remineralization strategies.

## 5. Conclusions

This study highlights the significant impact of different treatments on the microhardness of primary teeth. Sound primary teeth (Baseline group) exhibited the highest microhardness values, reflecting their natural structural resilience. In contrast, the control group, subjected to acid-induced artificial caries and stored in artificial saliva without treatment, showed the lowest microhardness, emphasizing the detrimental effects of demineralization and the insufficiency of artificial saliva alone to restore hardness.

Within the limitations of this in vitro study, SDF Advantage Arrest demonstrated significantly greater remineralization of artificial enamel lesions compared to SDF Riva Star Aqua and SDF Riva Star, which showed statistically similar results. The low within-group variability and clear statistical distinctions underscore the reliability of these findings, with Advantage Arrest achieving a higher mean microhardness than Riva Star Aqua and Riva Star. These results suggest that the formulation of SDF Advantage Arrest, potentially due to optimized silver ion availability or pH stability, provides enhanced remineralization and structural strengthening, positioning it as a promising option for managing early enamel lesions in primary teeth.

However, these findings should be interpreted with caution, given the in vitro setting, which lacks the dynamic oral environment, including biofilm and long-term aging effects. Well-designed clinical trials are essential to validate these results, assess in vivo efficacy, and establish evidence-based clinical guidelines for using SDF formulations in pediatric dentistry. Future research should also explore the mechanisms underlying Advantage Arrest’s superior performance to optimize remineralization strategies and preserve primary tooth integrity.

## Figures and Tables

**Table 1:** Composition of demineralization and remineralization solutions.

*Solution*	*Composition*
*Demineralization solution*	Calcium: 2.0 mmol/L Phosphates: 2.0 mmol/L Acetate: 75 mmol/L
*Artificial Saliva: Remineralization solution*	Na_2_HPO_4_: 2.38 g KH_2_PO_4_: 0.19 g NaCl: 8 g

**Table 2:** Study groups.

*Study Group*	*Treatment*
*I. Baseline*	15 sound primary molars without treatment (no artificial caries formation).
*II. SDF RIVA STAR AQUA^®^ 38%*	15 primary molars treated with SDF Riva Star Aqua^®^ 38% following artificial caries formation.
*III. SDF RIVA STAR^®^ 38%*	15 primary molars treated with SDF Riva Star^®^ 38% following artificial caries formation.
*IV. SDF Advantage Arrest^®^ 38%*	15 primary molars treated with SDF Advantage Arrest^®^ 38% following artificial caries formation.
*V. Control Group*	After artificial caries formation, 15 primary molars are stored in artificial saliva (no remineralization treatment).

**Table 3:** Mean microhardness values and statistical comparisons across study groups.

*Study Groups*	*N*	*Mean*	*Std. Deviation*	*Std. Error*	*95% Confidence Interval for Mean*		*Schiffe as MCT*
					*Lower Bound*	*Upper Bound*	
*I. Baseline*	15	469.32	1.01	0.26	468.60	470.04	g
*II. SDF Riva Star Aqua*	15	323.32	1.79	0.46	322.33	324.32	c
*III. SDF Riva Star*	15	322.38	1.97	0.51	321.29	323.47	c
*IV. SDF Advantage Arrest*	15	333.59	1.82	0.47	332.58	334.60	d
*V. Control*	15	192.56	2.24	0.58	191.33	193.80	a

**Table 4:** ANOVA results for microhardness differences among SDF treatment groups.

*ANOVA*					
*Microhardness*					
	Sum of Squares	df	Mean Square	F	Sig.
*Between Groups*	1159.93	2	579.96	167.73	0.000
*Within Groups*	145.23	42	3.46		
*Total*	1305.16	44			

**Table 5:** Scheffé post hoc pairwise comparisons of microhardness among SDF groups.

*Post Hoc Tests*						
*Multiple Comparisons*						
*(I) Group*		Mean Difference (I-J)	Std. Error	Sig.	95% Confidence Interval	
					Lower Bound	Upper Bound
*II*	III	0.94444	0.67900	0.388	−0.7787	2.6675
	IV	−10.26667 *	0.67900	0.000	−11.9898	−8.5436
*III*	II	−0.94444	0.67900	0.388	−2.6675	0.7787
	IV	−11.21111 *	0.67900	0.000	−12.9342	−9.4880
*IV*	II	10.26667 *	0.67900	0.000	8.5436	11.9898
	III	11.21111 *	0.67900	0.000	9.4880	12.9342

* The mean difference is significant at the 0.05 level.

**Table 6:** Homogeneous subsets of SDF groups based on microhardness (Scheffé analysis).

*Micro Hardness*			
*Scheffe*			
*Group*	N	Subset for Alpha = 0.05	
		1	2
*III*	15	322.3800	
*II*	15	323.3244	
*IV*	15		333.5911
*Sig.*		0.388	1.000

Means for groups in homogeneous subsets are displayed; harmonic mean sample size = 15.000.

## Data Availability

The original contributions presented in this study are included in the article. Further inquiries can be directed to the corresponding author.
